# Geological Substrates Shape Tree Species and Trait Distributions in African Moist Forests

**DOI:** 10.1371/journal.pone.0042381

**Published:** 2012-08-15

**Authors:** Adeline Fayolle, Bettina Engelbrecht, Vincent Freycon, Frédéric Mortier, Michael Swaine, Maxime Réjou-Méchain, Jean-Louis Doucet, Nicolas Fauvet, Guillaume Cornu, Sylvie Gourlet-Fleury

**Affiliations:** 1 UPR Biens et services des écosystèmes forestiers tropicaux (BSEF), Centre de coopération internationale en recherche agronomique pour le développement (CIRAD), Montpellier, France; 2 Laboratoire de foresterie des régions tropicales et subtropicales (FORTROP), Unité de gestion des ressources forestières et des milieux naturels (GRFMN), Gembloux Agro-Bio Tech (GxABT), Université de Liège (ULg), Gembloux, Belgique; 3 Bayreuth Centre of Ecology and Environmental Research (BayCEER), Department of Plant Ecology, University of Bayreuth, Bayreuth, Germany; 4 Smithsonian Tropical Research Institute (STRI), Balboa, Ancon, Panama; 5 School of Biological Sciences, University of Aberdeen, Aberdeen, United Kingdom; 6 Laboratoire Evolution et Diversité Biologique, Centre national de la recherche scientifique (CNRS), Université Paul Sabatier, Toulouse, France; University of Alberta, Canada

## Abstract

**Background:**

Understanding the factors that shape the distribution of tropical tree species at large scales is a central issue in ecology, conservation and forest management. The aims of this study were to (i) assess the importance of environmental factors relative to historical factors for tree species distributions in the semi-evergreen forests of the northern Congo basin; and to (ii) identify potential mechanisms explaining distribution patterns through a trait-based approach.

**Methodology/Principal Findings:**

We analyzed the distribution patterns of 31 common tree species in an area of more than 700,000 km^2^ spanning the borders of Cameroon, the Central African Republic, and the Republic of Congo using forest inventory data from 56,445 0.5-ha plots. Spatial variation of environmental (climate, topography and geology) and historical factors (human disturbance) were quantified from maps and satellite records. Four key functional traits (leaf phenology, shade tolerance, wood density, and maximum growth rate) were extracted from the literature. The geological substrate was of major importance for the distribution of the focal species, while climate and past human disturbances had a significant but lesser impact. Species distribution patterns were significantly related to functional traits. Species associated with sandy soils typical of sandstone and alluvium were characterized by slow growth rates, shade tolerance, evergreen leaves, and high wood density, traits allowing persistence on resource-poor soils. In contrast, fast-growing pioneer species rarely occurred on sandy soils, except for *Lophira alata*.

**Conclusions/Significance:**

The results indicate strong environmental filtering due to differential soil resource availability across geological substrates. Additionally, long-term human disturbances in resource-rich areas may have accentuated the observed patterns of species and trait distributions. Trait differences across geological substrates imply pronounced differences in population and ecosystem processes, and call for different conservation and management strategies.

## Introduction

Identifying the factors that shape species distributions is a central issue in ecology. Since species distribution patterns underlie community composition, diversity and ecosystem function, an understanding of the factors forming these patterns is necessary for projecting consequences of climate and land use changes, and for designing effective conservation and forest management strategies.

Many tropical tree species are differentially distributed with respect to environmental factors at continental, regional and local scales [1]–[8]. The most consistent patterns in tropical forests are regional correlations between species distribution and rainfall [1], [5], [9], [10] and soil factors or topography [2], [7], [11]. At local scales, species associations with topography and soil factors have been reported in tropical forests worldwide [3], [4], [6], [12]–[14]. Topography and soil texture are related to soil moisture availability, and at the same time soil nutrient availability varies with topography and climate, as well as with underlying geology and soil age, suggesting that soil resources play a major role in shaping distribution patterns of tropical tree species. However, an observed match between species distribution patterns and environmental factors may arise coincidentally from dispersal limitation [15], from correlated biotic factors such as pest pressure [16], or from past human disturbances [17], [18]. When studying the determinants of species distribution in tropical forests, the spatial structure of the data must therefore be considered.

Few studies have yet related tropical tree species distribution patterns at large scales with processes that potentially determine them [7], [18], [19]. A trait-based approach can help elucidate such processes [7], [18]. Environmental and historical factors may impose barriers to the establishment, survival and/or growth of individuals and filter out species that are not physiologically adapted and do not possess the adequate functional traits [20]. Plant functional traits have been shown to be related both to species performance within habitats and to species distribution across habitats [19], [21]–[25]. In the tropics, trait-based approaches have been successfully used to identify mechanisms explaining coexistence of tree species at the local scale [25], and species distribution patterns at much larger scales [7], [18], [19]. Additionally, trait values of dominant species determine ecosystem function [26], [27]. Patterns of trait distribution can therefore give insights into both the processes shaping species distribution patterns and the consequences for ecosystem function [28].

The aims of this study were (i) to assess the role of environmental and historical factors in shaping the distribution patterns of common tree species in the semi-evergreen forests of the northern Congo basin taking spatial auto-correlation into account; and (ii) to identify potential mechanisms (e.g. environmental filtering or historical factors) explaining distribution patterns through a trait-based approach. At regional scale, we expected tropical tree species with similar distribution patterns to converge in strategy because of establishment, survival and/or growth barriers imposed by environmental and/or historical factors.

## Materials and Methods

In this study, we used abundance data for 31 common tree species in 56,445 0,5-ha plots spread over more than 700,000 km^2^ at the northern edge of the Congo basin and combined them with the spatial variation of factors potentially driving the tree distribution patterns as well as with values for functional traits indicative of resource use strategy. This is one of the first quantitative data sets on tropical forest composition assembled at this scale.

### Study area

**Figure 1:**
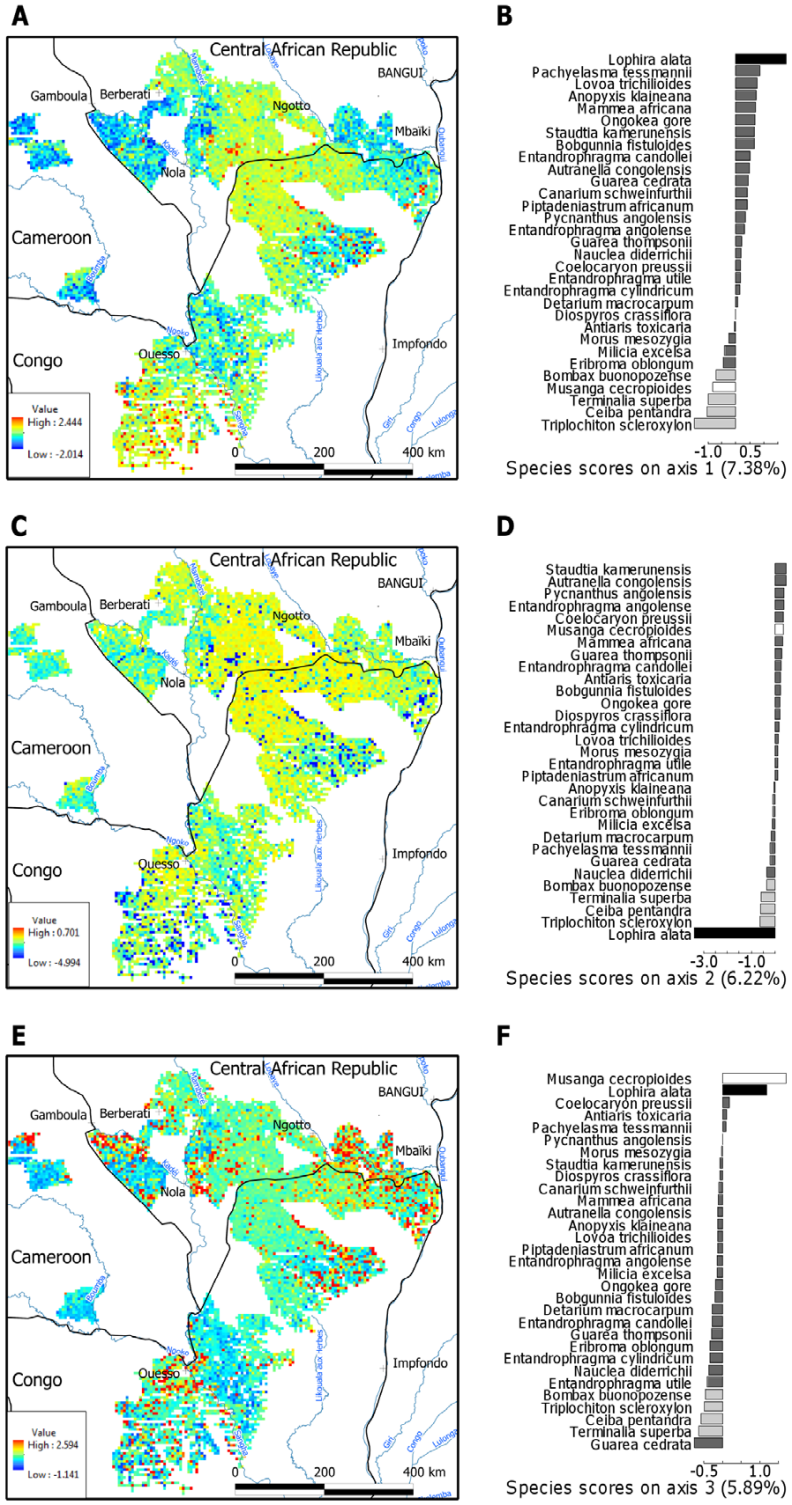
Geographical gradients of species composition and underlying patterns of species distribution. Plot scores on the first three compositional axes of a correspondence analysis of the plots (n = 56,445)×species (n = 31) abundance matrix were mapped (A, C, E). Variance explained by each axis is given in brackets. Solid lines represent country borders, names of main cities are indicated on the third map. Barplots give species scores across each compositional axis (B, D, F), with bar shading indicating the four species groups with contrasting distribution patterns that were identified by a cluster analysis (Fig. 2). Light and dark grey bars indicate species associated with the positive or negative end of the first compositional axis, respectively, while black and white bars indicate the two pioneer species, i.e. *Lophira* and *Musanga*, respectively, that both formed separate single-species groups.

The study area was distributed over south-eastern Cameroon, southern Central African Republic and northern Republic of Congo (Fig. 1). The extremes encompassed are 4°66′N and 0°34′N (northern and southern, 510 km apart); and 13°66′E and 18°62′E (western and eastern, 550 km apart). The vegetation consists of semi-evergreen rainforests of the Guineo-Congolian region.

### Abundance data

In the study area, forest inventories were conducted by logging companies to quantify timber resources and their spatial variation. Data from forest inventories have so far been little exploited in the Congo basin despite the consistency of protocols and the vast spatial area covered but see [29]. Commercial inventories are well-suited for identifying gradients of species composition and diversity because potential biases linked to poor identification are negligible for composition and diversity patterns [30]. We gathered data collected in 23 logging concessions (4, 8 and 11 in Cameroon, Central African Republic and Republic of Congo, respectively) over the period 2000–2007. All necessary permits were obtained for the described field studies. All inventories used a similar systematic sampling design, where all trees ≥30 cm diameter at breast height (dbh) were recorded in parallel transects (up to 69 km long) located 2–3 km apart from each other. Transects were divided into consecutive 0.5 ha plots (either 200 m×25 m or 250×20 m). Diameters up to 140 cm were assigned to 10 cm-wide dbh classes and larger trees were grouped together. Due to restricted access to data in five Congolian concessions, we used half of the inventoried plots (i.e. every other plot) for analyses throughout the study area (i.e. a total of 56,445 plots). Trees were identified to species level wherever possible. In this study, we focused on 31 common tree species, mostly timber species, which were reliably identified in the field. The species belong to 27 genera and 14 families, and encompass a large range of functional types (Table 1). Species nomenclature followed the work of Lebrun and Stork [31], and taxonomy was standardized according to the Angiosperm Phylogeny Group [32]. Throughout the text we first use full species names, and subsequently refer to the species by genus names for easier comparability with other tropical areas.

**Table 1:** Focal tree species, their overall abundance and frequency, and their functional traits.

Species (Family)	n	Freq (%)	Leaf pheno*	Shade tol†	WD (g.cm^−3^)	Growth (cm.yr^−1^)
*Anopyxis klaineana* (Rhizophoraceae)	7 783	11.49	Ever	P	0.725	0.796
*Antiaris toxicaria*. (Moraceae)	3 869	5.89	Deci	P	0.378	0.955
*Autranella congolensis* (Sapotaceae)	4 383	6.65	Deci	NPLD	0.782	1.273
*Bobgunnia fistuloides* (Fabaceae)	1 319	2.13	Ever	NPLD	0.876	0.700
*Bombax buonopozense* (Malvaceae)	1 29	2.05	Deci	P	0.382	1.114
*Canarium schweinfurthii* (Burseraceae)	2 71	4.39	Deci	P	0.418	1.114
*Ceiba pentandra* (Malvaceae)	5 262	7.46	Deci	P	0.275	1.910
*Coelocaryon preussii* (Myristicaceae)	14 133	16.28	Ever	ST	0.446	0.796
*Detarium macrocarpum* (Fabaceae)	2 385	3.77	Deci	P	0.565	1.114
*Diospyros crassiflora* (Ebenaceae)	11 56	15.28	Ever	ST	0.658	0.637
*Entandrophragma angolense* (Meliaceae)	8 383	12.68	Deci	NPLD	0.461	0.955
*Entandrophragma candollei* (Meliaceae)	7 133	11.10	Deci	NPLD	0.572	0.796
*Entandrophragma cylindricum* (Meliaceae)	28 271	33.80	Deci	NPLD	0.573	1.273
*Entandrophragma utile* (Meliaceae)	2 203	3.65	Deci	NPLD	0.521	0.955
*Eribroma oblongum* (Malvaceae)	14 923	18.95	Deci	ST	0.484	1.114
*Guarea cedrata* (Meliaceae)	6 755	9.25	Ever	ST	0.527	0.796
*Guarea thompsonii* (Meliaceae)	9 859	12.95	Ever	ST	0.568	0.637
*Lophira alata* (Ochnaceae)	9 807	9.48	Ever	P	0.864	0.637
*Lovoa trichilioides* (Meliaceae)	3 903	5.48	Deci	NPLD	0.440	1.353
*Mammea africana* (Clusiaceae)	5 624	8.16	Ever	ST	0.586	0.509
*Milicia excelsa* (Moraceae)	4 613	7.01	Deci	P	0.547	1.273
*Morus mesozygia* (Moraceae)	993	1.59	Deci	P	0.725	1.114
*Musanga cecropioides* (Urticaceae)	36 382	21.9	Ever	P	0.250	3.820
*Nauclea diderrichii* (Rubiaceae)	2 888	4.56	Ever	P	0.627	1.253
*Ongokea gore* (Olacaceae)	13 798	19.63	Ever	NPLD	0.715	0.955
*Pachyelasma tessmannii* (Fabaceae)	1 68	2.68	Deci	ST	0.614	0.780
*Piptadeniastrum africanum* (Fabaceae)	12 65	18.01	Deci	NPLD	0.587	2.324
*Pycnanthus angolensis* (Myristicaceae)	29 384	32.96	Ever	NPLD	0.414	1.114
*Staudtia kamerunensis* (Myristicaceae)	46 424	41.73	Ever	ST	0.744	0.637
*Terminalia superba* (Combretaceae)	36 743	30.34	Deci	P	0.450	2.228
*Triplochiton scleroxylon* (Malvaceae)	18 483	13.12	Deci	P	0.327	1.910

Total stem number (n) and frequency of occurrence (Freq, % of plot presences) were calculated in the 56,445 0.5-ha plots. Leaf phenology (Leaf pheno) and shade tolerance (Shade tol) were extracted from [36]–[38] and complemented by field observations (J.L. Doucet, pers. obs.). Wood density (WD, g.cm^−3^) and maximum growth rates (cm.yr^−1^) are from [39].

Abbreviations for leaf phenology and shade tolerance correspond to:

* Deci: deciduous species; Ever: evergreen species.

† P: pioneer species; NPLD: non-pioneer light demanding species; ST: shade tolerant species.

### Environment and human disturbance

#### Environmental factors

We assigned the values of five environmental variables pertaining to climate (annual rainfall and dry season length), topography (slope and altitude) and geology (8 distinct substrates) to each plot. Climatic data were obtained by spatial extrapolation of METEOSAT records (P. Mayaux, pers. com.). According to these data, mean annual rainfall varied between 1200 mm and 1700 mm over the study area. While the METEOSAT records tended to underestimate rainfall in comparison with available ground measurements, they were suitable for comparisons among sites. The climate in the study area is characterized by a pronounced dry season of varying duration. The length of the dry season (in months) with rainfall less than 50 mm was used to quantify the seasonality of rainfall, which ranged from 0 to 3 months. Topography was characterized by slope (0–112%) and altitude (270–1070 m), obtained from the Shuttle Radar Topography Mission (SRTM). A homogenized geological map of the study area was obtained through the comparative analysis of three national maps [33]–[35]. Eight distinct geological substrates were identified based on their origin (sedimentary, metamorphic, igneous) and chemical composition: (1) alluvium (24.3% of the plots), (2) sandstone (45.0%), (3) tillite (1.1%), (4) carbonate (all sedimentary rocks, 6.8%), (5) acid metamorphic rocks (schist and gneiss, 9.3%), (6) acid igneous rock (granite, 0.8%), (7) basic rocks (dolerite and amphibolite-facies, 3.5%), and (8) quartzite (a mixture of metamorphic and sedimentary quartzite-sandstone rocks, 9.2%).

#### Human disturbance

To assess recent human disturbance (i.e. in the 20^th^ century), we extracted information on land cover through digitalizing 15 topographical and land-use maps (1∶200 000 scale) by the French national mapping agency (IGN), published between 1959 and 1964 (maps are available in the IGN cartographic library, Paris, France). Most of the plots experienced no or minor human disturbances, and were classified as dense forest (97.2%), seasonally flooded forest (85 plots, 0.2%) or swamps (477 plots, 0.9%). The remaining plots were classified on the maps as degraded forest (180 plots, 0.3%) or savannas and plantations (843 plots, 1.5%), and we considered them as disturbed for the purpose of this study. Additionally, we included buffer zones in which forest plots were considered to have been prone to recent human disturbance: 15, 10, 5 and 3 km around main towns, main villages, secondary villages and hamlets respectively, and of 1 km and 500 m around main roads and tracks, respectively (6,780 plots, 12%). Combining information on land cover from old maps and on proximity to road and villages from recent maps (buffer zones), a total of 7,382 plots (13%) were classified as disturbed. For maps of environmental and historical factors see Figure S1.

### Species traits

We compiled information on four key functional traits of tropical trees: leaf phenology, shade tolerance, wood density and maximum growth rate. Information on leaf phenology was compiled from [36], [37] and complemented by field observations (J. L. Doucet, pers. obs.). Of the focal species, 42% were classified as evergreen, and 58% as deciduous (Table 1). Shade tolerance was characterized based on the species' regeneration guilds identified in Ghana [38]. Pioneers (P) require gaps for establishment (42% of the species), non-pioneer light-demanding species (NPLD, 32%) can establish in shade but need a gap to grow to their full height, and shade tolerant species (ST, 26%) can be found in shade both as young and older plants (shade-bearing guild in Hawthorne's terminology). Wood density was extracted from the database on wood technological properties assembled by CIRAD [39]. Values ranged from 0.25 to 0.88 g cm^−3^ among the focal species. Maximum annual diameter growth was calculated based on the 95^th^ percentile in 10 permanent 4-ha plots at the Mbaïki experimental station in Central African Republic [39]. Maximum diameter growth rate ranged between 0.50 and 3.82 cm yr^−1^.

### Statistical analyses

We investigated the main axes of variation in species composition and underlying patterns of species distribution with correspondence analysis (CA) of the plots (n = 56,445)×species (n = 31) abundance matrix. CA, also known as reciprocal averaging, produces a simultaneous ordination of plots and species [40]. It is a robust method for identifying compositional gradients within large datasets, while the dimensions of the abundance matrix did not allow the use of methods based on dissimilarities. Since we focussed on abundant species, the results were not influenced by correspondence between species-poor plots and rare species [41]. Plot scores resulting from the CA were used as a measure of community composition and species scores as a measure of species distribution across the respective axes. To explore spatial patterns of species composition, we mapped the plot scores for the first three axes. Using a hierarchical cluster analysis on the Euclidean distances between species scores and an average linkage, we identified groups of species according to their distribution patterns, i.e. their position across the compositional axes. We chose the average linkage (UPGMA) because it is a compromise between complete and single linkage that tends to produce compact clusters and single-species groups branched to large clusters, respectively. Complete linkage produced exactly the same results.

To identify the main determinants of species composition and patterns of species distribution, we first used linear models relating plot scores on the first three compositional axes to the set of explanatory variables pertaining to environment and history of human disturbances. As to be expected, the residuals of the linear models remained highly spatially autocorrelated for the three axes (with values of Moran's I of 0.23, 0.13 and 0.18, respectively). We then used simultaneous autoregressive (SAR) models to deal with spatial autocorrelation. We specifically used spatial error models, which are recommended for spatially autocorrelated distribution data [42]. In spatial error models, a term incorporating the spatial autocorrelation structure of the data is introduced in the standard linear regression model. The additional term is implemented with a spatial weights matrix defining the neighbourhood of each plot (distance to other plots) and the weight of each neighbour (depending on distance, closer plots having higher weights). SAR models take the following form:
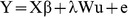
(1)where Y is the dependent variable (plot scores of the first three axes), X the explanatory variable (i.e. climate, geology and human disturbance), respectively, β is the coefficient associated with the explanatory variable, λ is the spatial autoregression coefficient, W is the spatial weights matrix, *u* is the spatially dependent error term and *e* represents the spatially independent error term. In this paper, we used the maximal distance of the k-nearest neighbours to define the neighbourhood of each plot. We selected k combining expert knowledge and the Bayesian Information Criterion (BIC). First, k should reflect the distance between two transects (2–3 km) so that two consecutive plots within a transect are strongly spatially correlated and that two close plots between transects should be more correlated than two distant plot within a transect. Second, we used the BIC to select among k = 1, k = 2, until k = 8. Best spatial models were found for k = 1 and a maximal distance of 4.5 km, that was in agreement with expert knowledge. The average number of neighbours was equal to 61.4, one plot had one neighbour and five plots had 129 neighbours.

Finally, to quantify the relative impact of each environmental and historical variable on the three compositional axes, we used a simple forward approach based on the Likelihood Ratio test (LR test) and the BIC to select explanatory variables sequentially [43]. We first used the LR test to assess the significance of adding a new explanatory variable to the null model, and the BIC increment to select the most important explanatory variable (lowest BIC) among significant explanatory variables (significant LR tests). Similarly, we used the LR test to assess the significance of adding a new explanatory variable to the model with one variable, and the BIC increment to select the most important additional explanatory variable.

To assess differences in species composition among geological substrates, we compared plot scores on the first compositional axis for pairs of substrates using pairwise Wilcoxon tests at P<0.001. Additionally, we analyzed differences in stand structure (i.e. total plot basal area) among substrates, to control for a possible confounding effect of stand age. We tested the relationship between species distribution patterns (species scores on the first compositional axis) and key functional traits. We used Kruskall-Wallis one-way analysis of variance for categorical traits (leaf phenology and shade tolerance) and Spearman correlation coefficients for quantitative traits (wood density and growth rate). We tested for significant differences at P<0.05 in species scores among shade tolerance categories using pairwise Wilcoxon tests. All analyses were performed within the R environment [44] using the following packages: ade4, fields and spdep [45]–[47].

## Results and Discussion

### Inventory data

The 31 focal species represented on average 17.5% of the stems and 25.3% of basal area in the plots. These common tree species occurred in immense areas of African rainforest, similar to the situation in upper Amazonian forests [48], [49]. Extremely large areas of distribution, sometimes covering the entire Guineo-Congolian domain, have long been recognized by foresters [9]. *Staudtia kamerunensis* (Myristicaceae) was the most abundant species with 46,424 trees inventoried (13.1% of the stems in the 31-species set) and was also the most frequent species (occurring in 41.7% of plots, Table 1). The next most abundant species, *Terminalia superba* (Combretaceae), *Musanga cecropioides* (Urticaceae), *Pycnanthus angolensis* (Myristicaceae), and *Entandrophragma cylindricum* (Meliaceae), accounted for 10.3, 10.2, 8.3 and 8.0% of the trees in the 31-species set, respectively.

### Spatial patterns of species composition

We identified three dominant and independent geographical gradients of tree species composition (Fig. 1), which together explained 20% of all variance, a value comparable to studies conducted elsewhere in the tropics [3], [7]. The first compositional axis (7.4% variance explained) highlighted two contrasting assemblages and patterns of species distribution. Species with extreme negative scores (Fig. 1B), were absent from a central zone in the study area and the most southern area but abundant elsewhere (in blue in Fig. 1A). The remaining species occurred across the whole study area but tended to reach high abundance in the central and southern areas (in yellow to red in Fig. 1A). The second and third compositional axes (6.2 and 5.9% variance explained respectively) were mostly driven by the remaining variations in the distribution of two pioneer species: *Lophira alata* (Ochnaceae, Fig. 1C and D) and the short-lived *Musanga* (Fig. 1E and F).

**Figure 2:**
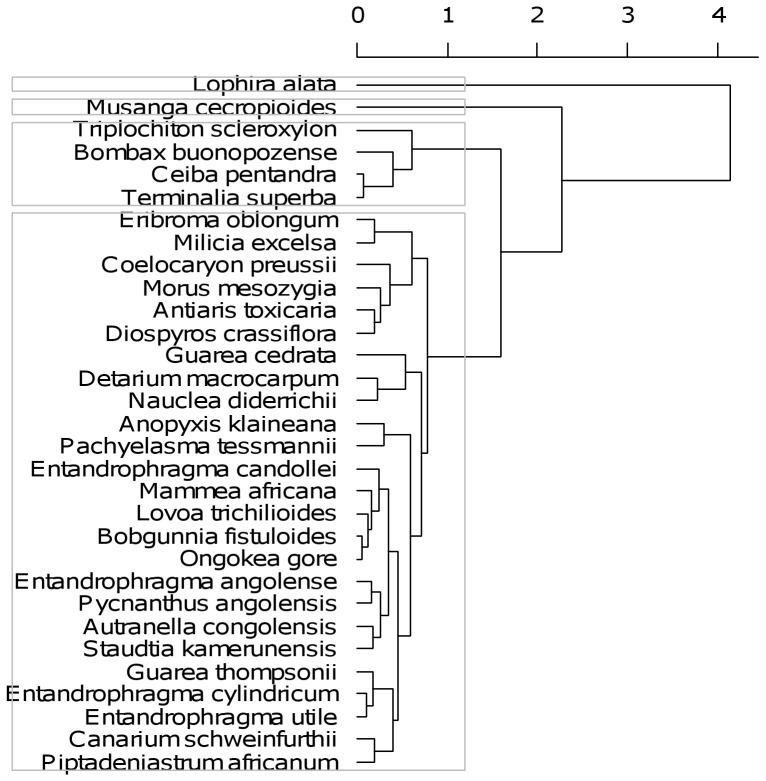
Cluster dendrogram grouping species with similar distribution patterns. A hierarchical cluster analysis on the Euclidean distances between species scores and an average agglomeration method was used to identify groups of species according to their distribution patterns, i.e. position across the compositional axes. The grey boxes indicate the cut-off level used to identify the four groups.

In agreement with the patterns described above, a cluster analysis of the species scores identified four contrasting distribution patterns (Fig. 2). *Lophira* and *Musanga* formed two single-species groups. A third group of species was composed of *Triplochiton scleroxylon*, *Ceiba pentandra*, *Bombax buonopozense* (all Malvaceae), and *Terminalia*, while the remaining 25 species encompassed a fourth group.

### Factors determining species distribution patterns

Geological substrate, climate and the recent history of human disturbance were the main drivers of species distribution patterns in the study area, which were strongly spatially autocorrelated (Table 2).

**Table 2:** Results of spatial regression models relating environmental and historical factors to compositional axes.

Model*	BIC	LR test	df	P-value	λ	P-value (λ)
Axis 1∼1	94 545					
Axis 1∼geology	94 529	−92.3	7	<2.2×10^−16^	0.90	<2.2×10^−16^
Axis 2∼1	123 664					
Axis 2∼dry season	123 575	−99.9	1	<2.2×10^−16^		
Axis 2∼dry season+geology	123 555	−97.0	7	<2.2×10^−16^	0.77	<2.2×10^−16^
Axis 3∼1	92 536					
Axis 3∼disturbance	92 463	−83.9	1	<2.2×10^−16^		
Axis 3∼disturbance+dry season	92 450	−23.8	1	0.4×10^−5^	0.83	<2.2×10^−16^

We used spatial error models to identify the most important factors (environment or history) for species composition taking spatial autocorrelation into account. To select explanatory variables sequentially we used a simple forward approach. At each step from the null model, we used the Likelihood Ratio test (LR test) to assess the significance of adding a new explanatory variable in the model and the Bayesian Information Criteria (BIC) to select the most important variable. Best spatial models (lowest BIC) are given for the three compositional axes. The value and significance of the spatial autoregression coefficient (λ) is also given for the best spatial models.

* For convenience, the spatial term (λW*u*) and coefficients (β) have been omitted in the model description (see Material and Methods for details).

#### Geology

**Figure 3:**
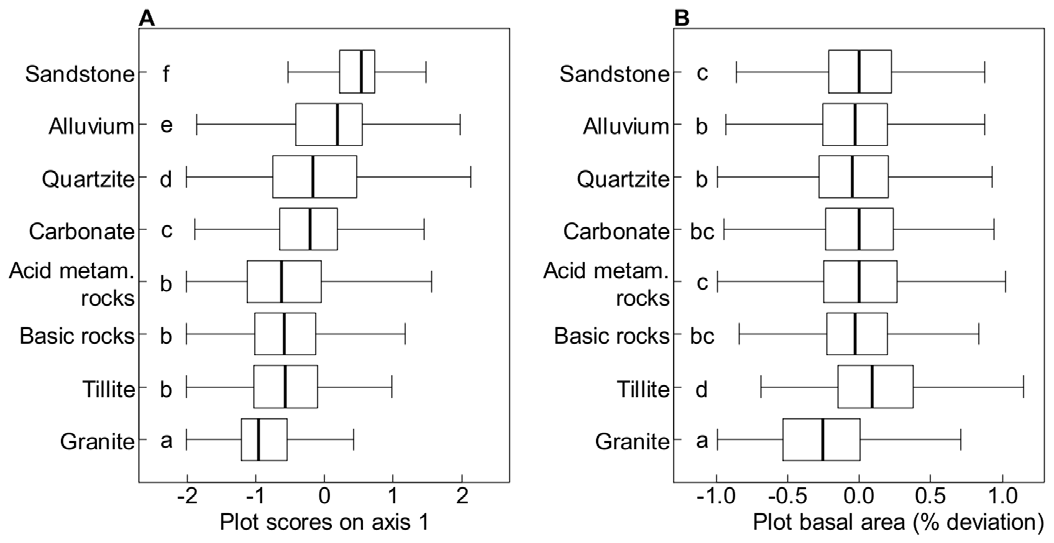
Compositional and structural differences among forests growing on different geological substrates. Plot scores on the first compositional axis were used to describe the main variation in species composition (A). Structural differences were assessed as the % deviation from mean plot basal area (B). Significant differences between substrates at P<0.001 in paired Wilcoxon tests are indicated by different letters.

Species distribution and community composition were strongly affected by the underlying geological substrate (Table 2). Species composition differed significantly between most geological substrates (in pairwise comparisons, Fig. 3A), with only three geological substrates sharing species composition (acid metamorphic rocks, basic rocks and tillite). Sandstone and, to a lesser degree, alluvium, were associated with positive scores on the first compositional axis, while the remaining geological substrates were all associated with negative plot scores. Differences in species composition among substrates were not associated with differences in stand structure, except on tillite and granite, where the values of plot basal area were higher and lower than average, respectively (Fig. 3B).

A large sandstone plateau covering ca. 25,300 km^2^ in the centre of the study area played a dominant role in shaping distribution patterns (Figs. 1 and S1). The importance of the sandstone plateau has previously been noted in southern Central African Republic [29]. Our analyses highlight its importance at a much larger spatial scale. Our results from the Congo basin are consistent with differences in species distribution and community composition among geological substrates reported in Amazonia [6], [7], [50]. The particular role of sandstone has been pointed out in Columbia, where sandstone plateaus are common landscape features [51], [52]. In this study, we focused on abundant and wide-spread species, excluding those with narrow occurrence and potentially more specific environmental requirements. It is therefore not unlikely that our results underestimate the extent to which geological substrate determines tree species distributions in the semi-evergreen forests of the northern Congo basin.

The strong association between tree species distributions and geological substrates, on which soils develop that have distinct chemical and structural properties and consequently different nutrient and water availability, suggests that soil resource availability is important for these patterns. Dominant soils on both the sandstone and alluvium substrates were characterized as sandy loam [53], while soils dominating the other substrates were mostly clay loam soils. Sandy soils are known to differ strongly from clay soils in resource availability: they retain neither nutrients nor water [54] and both nutrient and water availability have been shown to influence plant performance and species distribution patterns in tropical forests [2], [10], [12], [55]–[57]. Environmental filtering through low availability of nutrients and/or water may therefore exclude those species abundant on clay soils on a variety of geological substrates (the group composed of *Triplochiton*, *Ceiba*, *Bombax* and *Terminalia*, in grey in Fig. 1B and *Musanga*) from the sandy soils typical of sandstone and alluvium. On the other hand, specific adaptations allow another set of species (the group composed of the 25 species and *Lophira*) to grow and reach high abundance on the resource-poor soils.

Previous experimental and observational data on the two species with the most contrasting distribution patterns (*Triplochiton*, absent from sandstone, lowest score on the first compositional axis, and *Lophira*, abundant on sandstone, highest score), support their differential soil resource requirements, and the decisive role of nutrients in driving their distribution patterns. In experiments, high soil fertility strongly favoured the growth of *Triplochiton*, while the growth of *Lophira* was favoured by infertile soils, indicating an actual preference for low nutrient conditions in the latter species [57]. At the same time, *Lophira* is likely to be considerably more drought sensitive than *Triplochiton,* as suggested by their large scale distribution patterns across a pronounced rainfall (1000–3400 mm) in Western Africa [19], and by the use of *Lophira* as an indicator of wet conditions in paleoecological studies [58]. Thus, the absence of *Triplochiton* and the high abundance of *Lophira* on sandy soils is consistent with nutrient requirements, but not with drought sensitivity, in shaping species distribution patterns in the northern Congo basin.

#### Additional factors

In contrast to geology, climate and disturbance had significant but lesser influence on species distribution patterns, determining the remaining variations in the distribution of two pioneer species *Lophira* and *Musanga* (Fig. 1 and Table 2). The second compositional axis, driven by *Lophira*, was associated with a short dry season and the sandstone and alluvium substrates (Table 2). This result confirmed the affinity of *Lophira* for wet conditions. The third compositional axis, driven by the remaining variations in the distribution of the pioneers, *Musanga* and, to a lesser extent, *Lophira*, was associated with the recent history of human disturbances (around main roads and cities) in sites with a pronounced dry season (Fig. 1E and Table 2).

These results were in marked contrast with other studies showing strong relationships between tree species distribution and rainfall [1], [5], [9], [10] and were likely due to the considerably smaller variation of rainfall and dry season intensity in the study area compared to studies conducted in other regions. For example, in central Panama, where rainfall was found to be a significant predictor of species distributions, annual rainfall across the study area ranged from 1500 to 3600 mm [5], [10]. In contrast, in the present study, rainfall only varied from 1200 to 1700 mm across the study area.

Ancient human disturbances (>100 y, not accounted for in the disturbance variable used here) may have accentuated the observed patterns of species distributions across geological substrates. Specifically, forests growing on sandy soils may have experienced little human disturbances in the past because local people may have preferred fertile sites for slash and burn agriculture. Such differential land use would have favoured the abundance of fast growing pioneer species on fertile soils, and the persistence of slow growing species on less fertile sandy soils. Footprints of ancient human disturbances have indeed been demonstrated in tropical forests worldwide [17], [18], [59].

### Functional trait distributions

**Figure 4:**
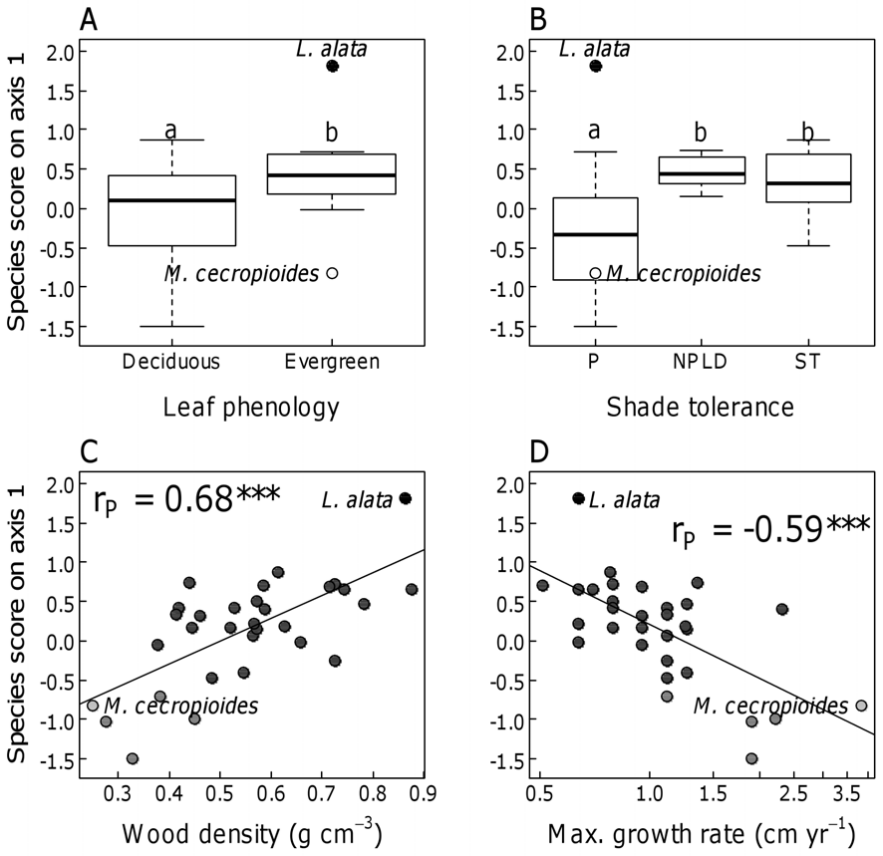
Functional differences among species associated with different geological substrates. Species scores on the first compositional axis were used as an indicator of species association with the geological substrate. Relationships between species scores and functional traits were assessed for leaf phenology (A), shade tolerance (B), wood density (C), and maximum annual growth rate (D). Different lower case letters above the boxplots indicate significant differences (P<0.05) in paired comparisons using Wilcoxon tests. Regression lines were plotted for quantitative traits. Symbol shading indicates the four species groups with contrasting distribution patterns: light and dark grey symbols indicate species positively or negatively associated with the sandstone substrate, respectively, while black and white symbols indicate the two pioneer species *Lophira* and *Musanga*.

We examined whether species with contrasting distribution patterns exhibited characteristic suites of functional traits. We found that species scores on axis one, indicating species association with geological substrates, differed between species with different traits (Fig. 4).

The four traits examined were highly correlated with each other (Table S1) and species associated with sandy soils typical of sandstone and alluvium (high scores) tended to show a common trait combination of evergreen leaves, high wood density and slow diameter growth, and were classified as non-pioneer or shade-tolerant species (Fig. 4). Species that preferentially occurred on the other substrates were deciduous, had low wood density and fast growth, and were pioneers, except *Lophira,* an evergreen pioneer with high wood density.

Evergreen leaves tend to be favoured under low soil fertility and/or high soil moisture conditions, because they limit the loss of nutrients, but may increase water demand through year-round activity [60]–[62]. The prevalence of evergreen species on sandy soils, therefore again suggests that nutrient rather than water availability plays a dominant role in determining trait and species distribution in the study area.

Wood density of tropical trees has been shown to decrease with both soil fertility and rainfall [7], [19], [63], [64]. High wood densities are associated with low diameter growth under resource poor conditions and additionally, are mechanistically related to low vulnerability to xylem embolism which allows survival under drought conditions [65]. Thus, both low nutrient and low water availability may favour species with high wood density on sandy soils.

Pioneer species, except *Lophira*, were significantly less associated with sandy soils than shade-tolerant or non-pioneer light demanding species. Lower turnover rates characterizing weakly productive forests in resource poor environments may contribute to the low abundance and diversity of pioneer species on sandy soils [66].

Species associated with sandy soils exhibited low potential growth rates. Traits that enable plants to exploit low-resource environments, such as dense wood and evergreen leaves (see above) are physiologically linked to low growth rates [67].

The differential distribution of the four correlated traits, which are indicative of the trait syndromes of rapid acquisition *vs.* efficient conservation of resources [67], support the decisive role of soil resources, especially nutrients, in shaping species distribution patterns in the area. The traits associated with sandy soils characterize tree species adapted to low-resource environments with conservative resource use, whereas the species on the remaining soils exhibit traits for rapid resource acquisition.

The data are also consistent with a resource-related demographic trade-off, where low mortality rates (associated with low growth rates) allow persistence on low-resource soils, while high growth rates (associated with high mortality rates) give species an advantage on high-resource soils [13], [68]. Resource related trade-offs have been shown to shape local habitat associations for tropical trees with respect to nutrients [13], [68], as well as with respect to light [69]. Our data suggest that such demographic trade-offs also shape large scale distribution patterns in tropical forests.

Overall, our results show that species with similar distribution patterns converge in strategy, suggesting that species that do not possess adequate functional traits are not able to survive, grow or reproduce in the community [20]. Our results thus indicate strong environmental filtering due to differential soil resource availability across geological substrates for large scale species composition in tropical forests. They thus add to the accumulating body of literature on environmental filtering at different scales and across different environmental gradients elsewhere in the tropics [19], [23], [25].

The observed trait distributions have consequences for ecosystem function, suggesting high biomass and carbon storage, but low productivity and turn-over rates of the forests on resource-poor sandy soils [39]. Moreover, the traits characterizing species typical of sandy soils, especially their slow growth rates, suggest that these large stretches of forests may be very slow in recovering from human or climate-induced disturbances. Such substrate-related differences in forest dynamics and function should be taken into account when designing conservation and forest management strategies.

## Supporting Information

Figure S1 Maps of environmental and historical factors. Spatial variation of five environmental factors: annual rainfall (A), dry season length (B), slope (C) and altitude (D) and geology (E), and one historical factor (recent human disturbance, F) were quantified from maps and satellite records. Climate and topography correspond to satellite records (METEOSAT and SRTM, respectively) while geology is a synthesis of three national maps. The recent history of disturbance combines information on forest cover from old maps with data on proximity to road and villages from recent maps (see Material and Methods for details). The black polygons indicate the limits corresponding to the inventory data.
(TIF)

Table S1 Results of the pairwise relationships between the four functional traits. To test the correlation between pairs of functional traits, we used Spearman correlation coefficient (r_S_) for quantitative traits, Kruskal-Wallis chi-squared test (K-W χ^2^) for a mix of a quantitative and a categorical trait; and chi-squared (χ^2^) test for categorical traits.
(DOCX)

## References

[1] Swaine MD (1996) Rainfall and soil fertility as factors limiting forest species distributions in Ghana. J Ecol 84: 419–428.

[2] Clark DB, Clark DA, Read JM (1998) Edaphic variation and the mesoscale distribution of tree species in a neotropical rain forest. J Ecol 86: 101–112.

[3] Webb CO, Peart DR (2000) Habitat associations of trees and seedlings in a Bornean rain forest. J Ecol 88: 464–478.

[4] Harms KE, Condit R, Hubbell SP, Foster RB (2001) Habitat associations of trees and shrubs in a 50-ha neotropical forest plot. J Ecol 89: 947–959.

[5] Pyke CR, Condit R, Aguilar S, Lao S (2001) Floristic composition across a climatic gradient in a neotropical lowland forest. J Veg Sci 12: 553–566.

[6] Phillips OL, Vargas PN, Monteagudo AL, Cruz AP, Chuspezans ME, et al (2003) Habitat association among Amazonian tree species: a landscape-scale approach. J Ecol 91: 757–775.

[7] ter Steege H, Pitman NCA, Phillips OL, Chave J, Sabatier D, et al (2006) Continental-scale patterns of canopy tree composition and function across Amazonia. Nature 443: 444–447. 17006512 10.1038/nature05134

[8] Tuomisto H, Ruokolainen K, Yli-Halla M (2003) Dispersal, environment, and floristic variation of western Amazonian forests. Science 299: 241–244. 12522248 10.1126/science.1078037

[9] Bongers F, Poorter L, Rompaey RSAR, Parren MPE (1999) Distribution of twelve moist forest canopy tree species in Liberia and Côte d'Ivoire: response curves to a climatic gradient. J Veg Sci 10: 371–382.

[10] Engelbrecht BMJ, Comita LS, Condit R, Kursar TA, Tyree MT, et al (2007) Drought sensitivity shapes species distribution patterns in tropical forests. Nature 447: 80–82. 17476266 10.1038/nature05747

[11] Tuomisto H, Ruokolainen K, Kalliola R, Linna A, Danjoy W, et al (1995) Dissecting amazonian biodiversity. Science 269: 63–66. 17787706 10.1126/science.269.5220.63

[12] John R, Dalling JW, Harms KE, Yavitt JB, Stallard RF, et al (2007) Soil nutrients influence spatial distributions of tropical tree species. P Natl Acad Sci USA 104: 864–869. 10.1073/pnas.0604666104 PMC1783405 17215353

[13] Russo SE, Davies SJ, King DA, Tan S (2005) Soil-related performance variation and distributions of tree species in a Bornean rain forest. J Ecol 93: 879–889.

[14] Palmiotto PA, Davies SJ, Vogt KA, Ashton MS, Vogt DJ, et al (2004) Soil-related habitat specialization in dipterocarp rain forest tree species in Borneo. J Ecol 92: 609–623.

[15] Hubbell SP, Foster RB (1986) Biology, chance, and history and the structure of tropical rain forest tree communities. In: Diamond JM, Case TJ, editors. Community Ecology. pp. 314–329.

[16] Fine PVA, Mesones I, Coley PD (2004) Herbivores promote habitat specialization by trees in Amazonian forests. Science 305: 663–665. 15286371 10.1126/science.1098982

[17] Clark DA, Clark DB (1995) Edaphic and human effects on landscape-scale distributions of tropical rain forest palms. Ecology 76: 2581–2594.

[18] ter Steege H, Hammond DS (2001) Character convergence, diversity, and disturbance in tropical rain forest in Guyana. Ecology 82: 3197–3212.

[19] Maharjan SK, Poorter L, Holmgren M, Bongers F, Wieringa JJ, et al (2011) Plant functional traits and the distribution of West African rain forest trees along the rainfall gradient. Biotropica 43: 552–561.

[20] Keddy PA (1992) Assembly and response rules - Two goals for predictive community ecology. J Veg Sci 3: 157–164.

[21] Wright IJ, Westoby M (2001) Understanding seedling growth relationships through specific leaf area and leaf nitrogen concentration: generalisations across growth forms and growth irradiance. Oecologia 127: 21–29. 28547166 10.1007/s004420000554

[22] Poorter L, Bongers F (2006) Leaf traits are good predictors of plant performance across 53 rain forest species. Ecology 87: 1733–1743. 16922323 10.1890/0012-9658(2006)87[1733:ltagpo]2.0.co;2

[23] Lebrija-Trejos E, Pérez-García EA, Meave JA, Bongers F, Poorter L (2010) Functional traits and environmental filtering drive community assembly in a species-rich tropical system. Ecology 91: 386–398. 20392004 10.1890/08-1449.1

[24] Wright SJ, Kitajima K, Kraft NJ, Reich PB, Wright IJ, et al (2010) Functional traits and the growth-mortality tradeoff in tropical trees. Ecology 91: 3664–3674. 21302837 10.1890/09-2335.1

[25] Kraft NJB, Valencia R, Ackerly DD (2008) Functional traits and niche-based tree community assembly in an Amazonian forest. Science 322: 580–582. 18948539 10.1126/science.1160662

[26] Grime JP (1998) Benefits of plant diversity to ecosystems: immediate, filter and founder effects. J Ecol 86: 902–910.

[27] Garnier E, Cortez J, Billes G, Navas M-L, Roumet C, et al (2004) Plant functional markers capture ecosystem properties during secondary succession. Ecology 85: 2630–2637.

[28] Lavorel S, Garnier E (2002) Predicting changes in community composition and ecosystem functioning from plant traits: revisiting the Holy Grail. Funct Ecol 16: 545–556.

[29] Réjou-Méchain M, Pélissier R, Gourlet-Fleury S, Couteron P, Nasi R, et al (2008) Regional variation in tropical forest tree species composition in the Central African Republic: an assessment based on inventories by forest companies. J Trop Ecol 24: 663–674.

[30] Réjou-Mechain M, Fayolle A, Nasi R, Gourlet-Fleury S, Doucet JL, et al (2011) Detecting large-scale diversity patterns in tropical trees: Can we trust commercial forest inventories? Forest Ecol Manag 261: 187–194.

[31] Lebrun JP, Stork AL (1991) Enumération des plantes à fleurs d'Afrique tropicale. Conservatoire et Jardin Botaniques de Geneve

[32] Angiosperm Phylogeny Group (2003) An update of the Angiosperm Phylogeny Group classification for the orders and families of flowering plants: APG II. Bot J Linn Soc 141: 399–436.

[33] Gazel J (1956) Carte géologique à 1∶1 000 000 du Cameroun. Planche 1-B-Sud

[34] Orstom (1963) Carte géologique à 1∶2 000 000 du Congo.

[35] Boulvert Y (1996) Etude géomorphologique de la République Centrafricaine: carte à 1/1000000 en deux feuilles Ouest et Est. Notice explicative n°110 ORSTOM Éditions, Paris. p.

[36] Aubréville A (1959) La flore forestière de la Côte d'Ivoire. 2nd ed. Nogent-sur-Marne, France: Centre Technique Forestier Tropical. p.

[37] Lebrun J, Gilbert G (1954) Une Classification écologique des foręts du Congo. Bruxelles, Belgique: Institut national pour l'étude agronomique du Congo Belge. p.

[38] Hawthorne WD (1995) Ecological profiles of Ghanaian forest trees. Oxford: Oxford Forestry Institute, Department of Plant Sciences, University of Oxford. 345 p.

[39] Gourlet-Fleury S, Rossi V, Réjou-Méchain M, Freycon V, Fayolle A, et al (2011) Environmental filtering of dense-wooded species controls aboveground biomass stored on nutrient-poor soils in African moist forests. J Ecol 99: 981–990.

[40] Hill MO (1974) Correspondence analysis - Neglected multivariate method. Appl Stat-J Roy St C 23: 340–354.

[41] Couteron P, Pelissier R, Mapaga D, Molino JF, Teillier L (2003) Drawing ecological insights from a management-oriented forest inventory in French Guiana. Forest Ecol Manag 172: 89–108.

[42] Kissling WD, Carl G (2007) Spatial autocorrelation and the selection of simultaneous autoregressive models. Global Ecol Biogeogr 17: 59–71.

[43] Anderson D (2008) Model Based Inference in the Life Sciences: A Primer on Evidence. 2nd ed. Springer. 184 p.

[44] R Development Core Team (2011) R: A language and environment for statistical computing. Available: http://www.R-project.org. Accessed 2011 Sep.

[45] Dray S, Dufour AB (2007) The ade4 package: implementing the duality diagram for ecologists. Journal of statistical software 22: 1–20.

[46] Furrer R, Nychka D, Sain S (2012) fields: Tools for spatial data. Available: http://CRAN.R-project.org/package=fields. Accessed 2012 Mar.

[47] Bivand R, with contributions by Altman M, Anselin L, Assunção R, Berke O, Bernat A, et al. (2011) spdep: Spatial dependence: weighting schemes, statistics and models. Available: http://CRAN.R-project.org/package=spdep. Accessed 2012 Mar.

[48] Pitman NCA, Terborgh JW, Silman MR, Núñez VP, Neill DA, et al (2001) Dominance and distribution of tree species in upper Amazonian terra firme forests. Ecology 82: 2101–2117.

[49] Pitman NCA, Terborgh J, Silman MR, Núñez VP (1999) Tree species distributions in an upper Amazonian forest. Ecology 80: 2651–2661.

[50] Tuomisto H, Poulsen AD, Ruokolainen K, Moran RC, Quintana C, et al (2003) Linking floristic patterns with soil heterogeneity and satellite imagery in Ecuadorian Amazonia. Ecol Appl 13: 352–371.

[51] Duivenvoorden JE (1995) Tree species composition and rain forest-environment relationships in the middle Caquetá area, Colombia, NW Amazonia. Plant Ecol 120: 91–113.

[52] Arbelaez MV, Duivenvoorden JF (2004) Patterns of plant species composition on Amazonian sandstone outcrops in Colombia. J Veg Sci 15: 181–188.

[53] Boulvert Y (1983) Carte pédologique de la République Centrafricaine, à 1∶1,000,000. Notice explicative 100.

[54] Vitousek PM, Sanford RL (1986) Nutrient cycling in moist tropical forest. Annu Rev Ecol Evol S 17: 137–167.

[55] Hall JS, McKenna JJ, Ashton PMS, Gregoire TG (2004) Habitat characterizations underestimate the role of edaphic factors controlling the distribution of Entandrophragma. Ecology 85: 2171–2183.

[56] Comita LS, Engelbrecht BMJ (2009) Seasonal and spatial variation in water availability drive habitat associations in a tropical forest. Ecology 90: 2755–2765. 19886485 10.1890/08-1482.1

[57] Veenendaal EM, Swaine MD, Lecha RT, Walsh MF, Abebrese IK, et al (1996) Responses of West African forest tree seedlings to irradiance and soil fertility. Funct Ecol 10: 501–511.

[58] Brncic TM, Willis KJ, Harris DJ, Washington R (2007) Culture or climate? The relative influences of past processes on the composition of the lowland Congo rainforest. Philos T R Soc B 362: 229–242. 10.1098/rstb.2006.1982 PMC2311427 17255032

[59] Willis KJ, Gillson L, Brncic TM (2004) How “virgin” is virgin rainforest? Science 304: 402–403. 15087539 10.1126/science.1093991

[60] Aerts R (1995) The advantages of being evergreen. Trends Ecol Evol 10: 402–407. 21237084 10.1016/s0169-5347(00)89156-9

[61] Reich PB, Walters MB, Ellsworth DS (1997) From tropics to tundra: Global convergence in plant functioning. P Natl Acad Sci USA 94: 13730–13734. 10.1073/pnas.94.25.13730 PMC28374 9391094

[62] Givnish TJ (2002) Adaptive significance of evergreen vs. deciduous leaves: solving the triple paradox. Silva Fennica 36: 703–743.

[63] Muller-Landau HC (2004) Interspecific and inter-site variation in wood specific gravity of tropical trees. Biotropica 36: 20–32.

[64] Chave J, Coomes D, Jansen S, Lewis SL, Swenson NG, et al (2009) Towards a worldwide wood economics spectrum. Ecol Lett 12: 351–366. 19243406 10.1111/j.1461-0248.2009.01285.x

[65] Hacke UG, Sperry JS, Pockman WT, Davis SD, McCulloh KA (2001) Trends in wood density and structure are linked to prevention of xylem implosion by negative pressure. Oecologia 126: 457–461. 28547229 10.1007/s004420100628

[66] Phillips OL, Hall P, Gentry AH, Sawyer SA, Vásquez R (1994) Dynamics and species richness of tropical rain forests. P Natl Acad Sci USA 91: 2805–2809. 10.1073/pnas.91.7.2805 PMC43459 11607468

[67] Chapin FS, Autumn K, Pugnaire FI (1993) Evolution of suites of traits in response to environmental stress. Am Nat 142: S78–92.

[68] Russo SE, Brown P, Tan S, Davies SJ (2008) Interspecific demographic trade-offs and soil-related habitat associations of tree species along resource gradients. J Ecol 96: 192–203.

[69] Walters MB, Reich PB (1996) Are shade tolerance, survival, and growth linked? Low light and nitrogen effects on hardwood seedlings. Ecology 77: 841–853.

