# Supplementary figures and images for: Geological Substrates Shape Tree Species and Trait Distributions in African Moist Forests
Source: PLoS One. 2012 Aug 15;7(8):e42381. doi: 10.1371/journal.pone.0042381 (PMC3419707; doi:10.1371/journal.pone.0042381)

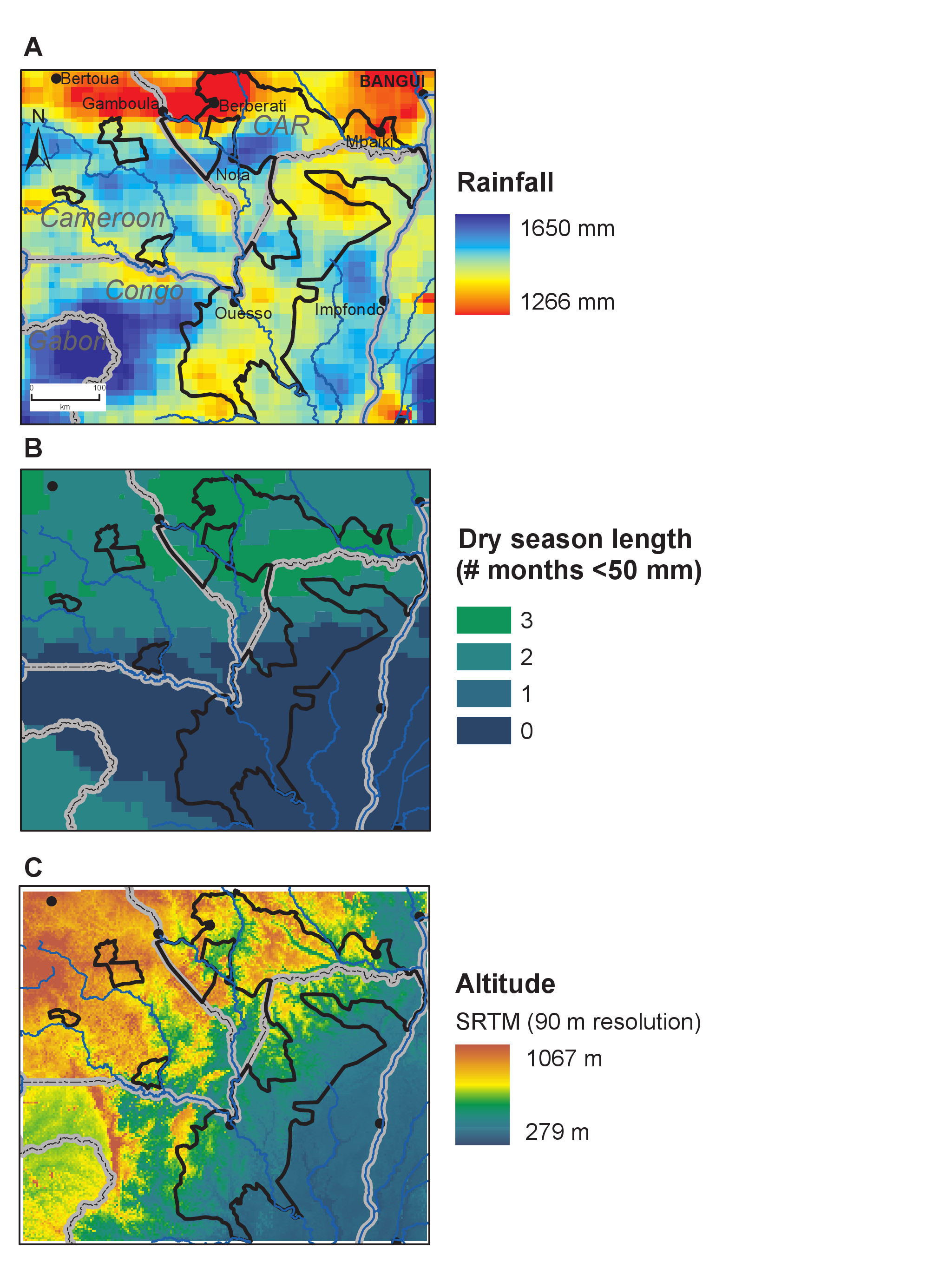

Supplement: Figure S1 — Maps of environmental and historical factors. Spatial variation of five environmental factors: annual rainfall (A), dry season length (B), slope (C) and altitude (D) and geology (E), and one historical factor (recent human disturbance, F) were quantified from maps and satellite records. Climate and topography correspond to satellite records (METEOSAT and SRTM, respectively) while geology is a synthesis of three national maps. The recent history of disturbance combines information on forest cover from old maps with data on proximity to road and villages from recent maps (see Material and Methods for details). The black polygons indicate the limits corresponding to the inventory data. (TIF) [file pone.0042381.s001.tif]
