# Supplementary material for: Geological Substrates Shape Tree Species and Trait Distributions in African Moist Forests
Source: PLoS One. 2012 Aug 15;7(8):e42381. doi: 10.1371/journal.pone.0042381 (PMC3419707; doi:10.1371/journal.pone.0042381)
Supplement: Table S1 — Results of the pairwise relationships between the four functional traits. To test the correlation between pairs of functional traits, we used Spearman correlation coefficient (rS) for quantitative traits, Kruskal-Wallis chi-squared test (K-W χ2) for a mix of a quantitative and a categorical trait; and chi-squared (χ2) test for categorical traits. (DOCX) [file pone.0042381.s002.docx]

Table S1

|  | Max. growth rate | Wood density | Leaf phenology |
| --- | --- | --- | --- |
| Wood density | r_S_ = -0.49 |  |  |
|  | *p = 0.005* |  |  |
| Leaf phenology | K-W χ² = 8.8 | K-W χ² = 3.5 |  |
|  | df = 1 | df = 1 |  |
|  | *p = 0.003* | *p = 0.063* |  |
| Shade tolerance | K-W χ² = 11.1 | K-W χ² = 2.4 | χ² = 4.8 |
|  | df = 2 | df = 2 | df = 2 |
|  | *p = 0.004* | *p = 0.295* | *p = 0.089* |
